# 850. Reasons for not Using PrEP and Actions that May Facilitate PrEP Uptake in Ontario and British Columbia, Canada

**DOI:** 10.1093/ofid/ofab466.1045

**Published:** 2021-12-04

**Authors:** Oscar Javier Pico Espinosa, Mark Hull, Nathan Lachowsky, David Hall, Saira Mohammed, Karla Fisher, Daniel Grace, Mark Gaspar, Robinson Truong, Leo Mitterni, Matthew Harding, Paul MacPherson, Kevin Woodward, Simon Rayek, Eric Peters, Jody Jollimore, Marshall Kilduff, John Maxwell, Warren Greene, Garfield Durrant, Camille Arkell, Tyllin Cordeiro, Darrell Tan

**Affiliations:** 1 St. Michael’s Hospital, Unity Health Toronto, Toronto, Ontario, Canada; 2 BC Centre for Excellence in HIV/AIDS, Vancouver, British Columbia, Canada; 3 University of Victoria, Victoria, British Columbia, Canada; 4 Vancouver Coastal Health, Vancouver, British Columbia, Canada; 5 Toronto General Hospital, Toronto, Ontario, Canada; 6 University of Toronto, Toronto, Ontario, Canada; 7 Hassle Free Clinic, Toronto, Ontario, Canada; 8 MAX Ottawa, Ottawa, Ontario, Canada; 9 University of Ottawa, Ottawa, Ontario, Canada; 10 McMaster University, Hamilton, Ontario, Canada; 11 Health Initiative for Men, Vancouver, British Columbia, Canada; 12 The Gay Men’s Sexual Health Alliance, Toronto, Ontario, Canada; 13 Community-Based Research Centre, Vancouver, British Columbia, Canada; 14 AVI Health and Community Services, Victoria, British Columbia, Canada; 15 AIDS Committee of Toronto, Toronto, Ontario, Canada; 16 Canadian Aboriginal AIDS Network, Fort Qu’Appelle, Saskatchewan, Canada; 17 Black Coalition for AIDS Prevention, Toronto, Ontario, Canada; 18 Canadian AIDS Treatment Information Exchange (CATIE), Toronto, Ontario, Canada; 19 Alliance for South Asian AIDS Prevention (ASAAP), Toronto, Ontario, Canada; 20 Division of Infectious Diseases, Department of Medicine, St. Michael’s Hospital, Toronto, Ontario, Canada

## Abstract

**Background:**

HIV Pre-exposure prophylaxis (PrEP) is an underutilized intervention to prevent HIV infection in Canada. Known barriers to PrEP uptake include lack of awareness, low HIV risk perception, side effects, PrEP not being publicly funded (which is the case in Ontario) and stigma. We aimed to identify barriers to PrEP use and actions that may facilitate PrEP uptake in Ontario and British Columbia.

**Methods:**

Gay, bisexual and other men who have sex with men 19 years or older living in Ontario and British Columbia, Canada, answered a survey between July 2019 and August 2020. Participants who met Canadian PrEP guideline criteria for PrEP and not already using PrEP indicated which barriers were relevant to them and which actions would make them more likely to start PrEP. We used descriptive statistics and tested differences between Ontario and British Columbia using Chi-square tests for proportions and t-tests or Wilcoxon rank-sum tests for continuous variables.

**Results:**

Of 1527 survey responses, 260 (184 in Ontario and 76 in British Columbia) who were never PrEP users and met criteria for PrEP were included. In Ontario, the most common barriers were affordability (43%) and concern about side effects (42%). In British Columbia, the most common reasons were concern about side effects (41%) and not feeling at high enough risk (36%). In Ontario, the actions that would most likely encourage the respondent to start PrEP were short waiting time (63%), the healthcare provider informing about their HIV risk being higher than perceived (62%) and a written step-by-step guide (60%). In British Columbia, the actions that would most likely encourage the respondent to start PrEP were short waiting time (68%), people speaking publicly about PrEP (68%) and their healthcare provider counselling about: their HIV risk being higher than perceived (64%), side effects of PrEP (64%) and about how PrEP works (62%).

Table. Top reasons for not using PrEP and top actions that might influence the decision to start PrEP stratified by province. (n= 184 in Ontario, n= 76 in British Columbia).

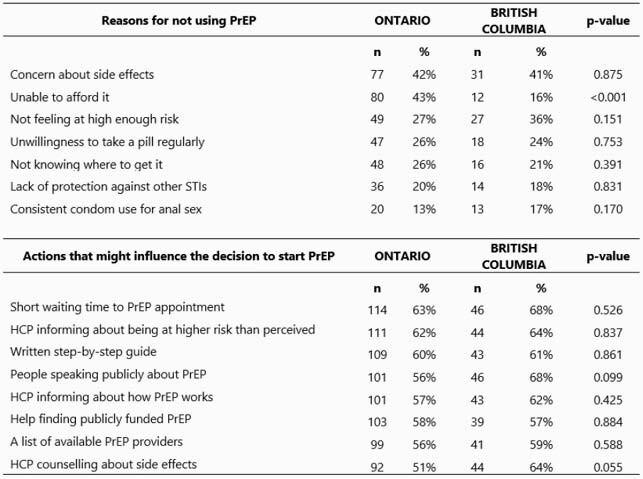

**Conclusion:**

Concern about side effects and not feeling at high enough risk were common barriers. Short waiting times may increase PrEP uptake. In Ontario, the findings suggested lack of affordability. In British Columbia, actions involving healthcare providers were valued.

**Disclosures:**

**Kevin Woodward, MD FRCPC**, **Gilead** (Independent Contractor) **Darrell Tan, MD PhD**, **Abbvie** (Grant/Research Support)**Gilead** (Grant/Research Support)**GlaxoSmithKline** (Scientific Research Study Investigator)**ViiV Healthcare** (Grant/Research Support)

